# Renal fat fraction is significantly associated with the risk of chronic kidney disease in patients with type 2 diabetes

**DOI:** 10.3389/fendo.2022.995028

**Published:** 2022-09-30

**Authors:** Yan Shen, Lianghua Xie, Xiangjun Chen, Lina Mao, Yao Qin, Rui Lan, Shumin Yang, Jinbo Hu, Xue Li, Hanwen Ye, Wenjin Luo, Lilin Gong, Qifu Li, Yun Mao, Zhihong Wang

**Affiliations:** ^1^ Department of Endocrinology, The First Affiliated Hospital of Chongqing Medical University, Chongqing, China; ^2^ Department of Radiology, The First Affiliated Hospital of Chongqing Medical University, Chongqing, China

**Keywords:** renal fat fraction, chronic kidney disease, ectopic fat deposition, type 2 diabetes mellitus, magnetic resonance imaging

## Abstract

**Backgrounds:**

Ectopic fat deposition is closely related to chronic kidney disease (CKD). Currently, there are few population studies that have been conducted to determine the relationship between renal parenchyma fat deposition and the risk of CKD among patients with type 2 diabetes mellitus (T2DM). Therefore, we employed magnetic resonance imaging (MRI) to detect renal parenchyma fat content in individuals with T2DM, expressed as renal fat fraction (FF), to explore whether renal FF is an important risk factor for CKD in patients with T2DM.

**Methods:**

In this cross-sectional study, 189 subjects with T2DM were enrolled. CKD was defined as the estimated glomerular filtration rate (eGFR)<60 mL/min/1.73m^2^. Measurement of the renal FF was performed on a 3.0-T MRI (MAGNETOM Skyra, Siemens, Erlangen, Germany). Binary logistic regression was used to determine the association between tertiles of renal FF and risk of CKD. Receiver-operator characteristic (ROC) curves were constructed to evaluate the sensitivity and specificity of renal FF in detecting CKD in T2DM patients.

**Results:**

The patients were divided into three groups according to tertiles of the renal FF level (2.498 - 7.434). As renal FF increases, patients tend to be older, and more abdominally obese, with a decreased eGFR (p<0.05). After adjustment for potential confounders, patients in the highest tertile of renal FF had a significantly increased risk of CKD than those in the lowest tertile (odds ratio (OR) = 3.98, 95% confidence interval (CI) = 1.12 - 14.09, p = 0.032), and the area under the ROC curve for this model was 0.836 (0.765–0.907).

**Conclusions:**

The renal FF is significantly independently associated with CKD in patients with T2DM.

## Introduction

Chronic kidney disease (CKD) is highly prevalent in patients with type 2 diabetes mellitus (T2DM), approximately 30%-50% patients with T2DM have CKD (1–4). CKD increases the risk of cardiovascular disease (5) and is the most common cause of end-stage renal disease (ESRD) worldwide (6), especially in patients with diabetes mellitus (DM) (7). The age-standardized 10-year cumulative all-cause mortality in patients with T2DM and CKD was increased by 24.4% (8). Due to severe complications and high mortality resulting from CKD, early identification of high-risk individuals with T2DM is a crucial strategy to prevent the development of CKD.

Patients with T2DM are often accompanied by lipid metabolism disorders (9), which are manifested in excess lipids that are ectopically deposited in non-adipose such as the liver, heart, pancreas, and kidney (10, 11). In the Kidney, ectopic fat often deposits in the perirenal space, renal sinus, and renal parenchyma (12). Previous studies (13–15) have confirmed that perirenal and renal sinus fat deposition is closely related to the increased risk of CKD, but the relationship between renal parenchymal fat deposition and CKD in patients with T2DM remains unclear.

Renal parenchymal fat deposition is a condition in which ectopic fat is deposited in the kidney’s cortex and medulla, which leads to renal cell injuries, such as podocytes, tubular cells, and mesangial cells (16–19). Numerous animal studies (20, 21) have shown that renal parenchymal lipid deposition causes kidney damage, such as glomerulosclerosis, renal interstitial fibrosis, and proteinuria. However, due to the limited methods of measuring renal parenchymal fat, which mainly rely on renal biopsy, invasive and complex, there have been few population studies of renal parenchymal fat deposition and renal impairment previously. Recently, Reeder SB et al. (22) used the proton-density fat fraction (FF) measured by magnetic resonance imaging (MRI) to evaluate the tissue fat deposition content, which is non-invasive, accurate, and convenient. With this method, Wang et al. (23) studied the relationship between renal parenchymal fat deposition and T2DM in 95 participants, and they found that the renal FF is significantly increased in diabetic patients, especially in patients with microalbuminuria. However, this research has not adjusted the other confounders, whether renal FF is independently associated with microalbuminuria is not clear. Besides, the impact of renal FF on renal function has not been reported.

In the current study, we measured fat deposited in renal parenchyma by MRI in patients with T2DM, to explore whether renal FF was an independent risk factor for CKD.

## Research design and methods

### Population

**Figure 1:**
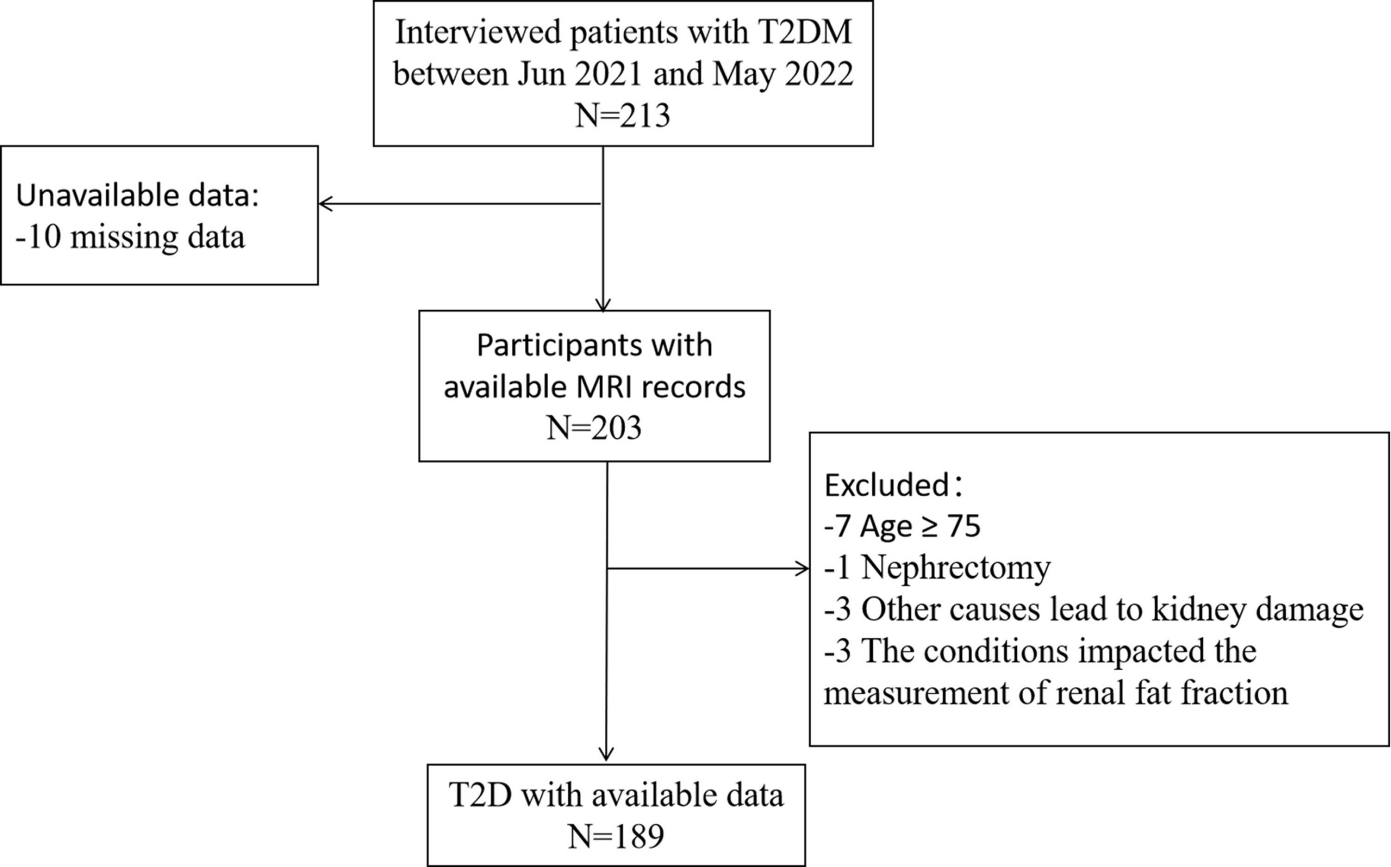
Flow chart of study population in the study.

We recruited 213 patients with T2DM who attended the Department of Endocrinology at the First Affiliated Hospital of Chongqing Medical University from June 2021 to May 2022. T2DM was diagnosed based on the 1999 World Health Organization diagnostic criteria for T2DM (24). All patients signed informed consent forms and completed an abdominal MRI scan. The exclusion criteria are as follows: 1) age ≥75 years; 2) a previous history of chronic kidney disease caused by hypertension, kidney stones, IgA nephropathy, etc; 3) severe liver damage (transaminases exceeding the upper limit of normal values more than 3 times) and severe heart failure (New York Heart Association cardiac function grades II-IV); 4) combined with renal tumors, renal large cysts, abnormal renal location, congenital dysplasia of the kidneys, and other conditions that affect the renal fat measurement. In total, 189 participants were involved in the final analyses. This study was supported by the Ethics Committee of The First Affiliated Hospital of Chongqing Medical University (Approval number: 2021-685). The research flowchart is shown in **Figure 1**.

### Data collection and biochemical measurement

Those who participated answered questions (about smoking, drinking, and their history of serious illness), and had their blood pressure, height, weight, waist circumference (WC) and hip circumference (HIP) measured. BMI was calculated as weight in kilograms divided by the square of height in meters.

Glycosylated hemoglobin (HbA1c) was measured by a high-performance liquid chromatography analyzer. Fasting plasma glucose (FPG), serum lipid levels such as total cholesterol (TC), triglyceride (TG), high-density lipoprotein cholesterol (HDL-C), and low-density lipoprotein cholesterol (LDL-C) were measured enzymatically by an automatic analyzer (Model 7080; Hitachi, Tokyo, Japan) with reagents purchased from Leadman Biochemistry Co. Ltd. (Beijing, China). Serum creatinine, urinary creatinine, and albumin were measured with an automatic biochemical analyzer (Modular DDP; Roche). The urinary albumin–to–creatinine ratio (UACR) was calculated.

### Definition of variables

Participants who self-reported having smoked or are currently smoking were defined as having a history of smoking. Drinking history was defined as patients who consumed ≥ 50 g of alcohol per day. Hypertension was defined as systolic blood pressure ≥ 140 mmHg, diastolic blood pressure ≥ 90 mmHg, or taking antihypertensive medications. The estimated glomerular filtration rate (eGFR) was calculated according to the Chronic Kidney Disease Epidemiology Collaboration (CKD-EPI) equation (25). CKD was defined as eGFR < 60 mL/min/1.73m^2^ (26).

### Measurement of PDFF

All MRI examinations were performed on a 3.0-T MRI system (MAGNETOM Skyra, Siemens, Erlangen, Germany) with a sixteen-channel phased-array body coil. The sequences main including T1 VIBE two-point Dixon sequence and multi-echo Dixon VIBE sequence with two consecutive breath holds. The parameters of T1 VIBE two-point Dixon sequence was as follows: repetition time (TR), 4.66ms; echo times (TEs), 1.34 and 2.57ms; slice thickness,3 mm; slice number, 64; matrix size, 210 × 320; number of excitations, 1; field of view (FOV), 400-450 mm ×87.5%; band-width, 820 Hz/pixel; acquisition time, 17 s. The multi-echo Dixon VIBE sequence as follows: TR, 9.46ms; TEs, 1.33, 2.64, 3.95, 5.26, 6.57, and 7.88ms; slice thickness,4 mm; slice number, 64; matrix size, 137 × 224; number of excitations, 1; FOV, 360-450 mm × 87.5%; band-width, 1060 Hz/pixel; acquisition time, 20 s. FF and Fat only maps were generated automatically after the acquisition using Siemens built-in post-processing toolkit.

**Figure 2:**
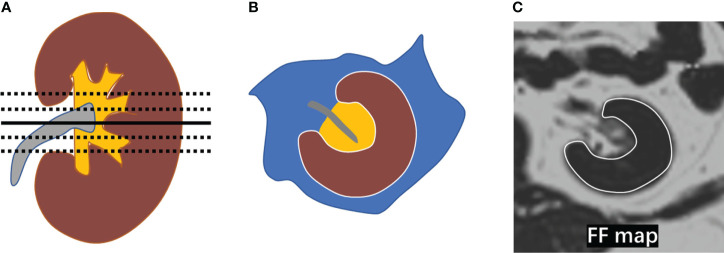
Measurement of renal FF. **(A)** The five levels (black line) centered on the renal hilum (black solid line) were selected in the kidney; **(B)** A diagram depicting the placement of a region of interest (ROI) in the entire renal parenchyma (brown area) with the boundary marked by a white line; **(C)** ROIs were manually placed on the five selected levels in the FF map avoiding the perirenal fat and renal sinus fat respectively.

The measurements of the FF were performed in a software (sygno.via, Siemens Healthcare, Erlangen, Germany). The five levels centered on the renal hilum on each side of the kidney were chosen respectively. The free-hand region of interest (ROI) was placed in the entire renal parenchyma with the boundaries avoiding the perinephric and renal sinus fat (**Figure 2**). The unilateral renal FF was calculated by the arithmetic mean value of the five sections of measurements, and renal FF was obtained by the sum of bilateral renal FF in each individual.

Two radiologists from the radiology department (reader A, L.H.X., 3 years of experience, and reader B, P.Y., 2 years of experience) performed data measurements and were blinded to all clinical data under the supervision of an experienced abdominal radiologist (Y.M., 18 years of experience). The consistency between two readers was analyzed using the intraclass correlation coefficient (ICC). For the agreement analysis, the ICC values was interpreted as follows: less than 0.5, poor reliability; 0.5 and 0.75, moderate reliability; 0.75 and 0.9, good reliability; greater than 0.90, excellent reliability (27). There was good or excellent reliability for renal FF between the two readers (ICC = 0.822).

### Statistical analyses

Statistical analyses were performed using SPSS 25.0 (IBM, Armonk, NY, USA). Continuous variables were described using mean ± standard deviation (SD) or median (25th and 75th percentiles), depending on whether the data distribution was normal (assessed by the Shapiro-Wilk test). As for Continuous variables, the Student t-test or Mann-Whitney U test was used to determine differences between two groups; ANOVA or the Kruskal-Wallis test was used to determine the differences among three groups. Categorical variables were expressed as frequencies(percentage), and x^2^ tests were used for group comparisons.

Binary logistic regression analysis was used to describe the relationship between renal FF and CKD while adjusting for potential confounding variables. Data were summarized as odds ratios and regression coefficients (OR, 95% CI). P < 0.05 was considered statistically significant.

## Results

### Clinical characteristics of the participants

A total of 189 patients with T2DM were included in the analysis. The clinical characteristics of participants grouped by three tertiles of the renal FF were shown in **Table 1**. The average age of the total population included was 51 years, with 66.1% of men. There were 85 (45.0%) patients who reported a history of smoking, while 55 (29.1%) had a history of alcohol drinking. As the renal FF increases, patients tend to be older, and more abdominally obese, with a decreased eGFR (p<0.05). There was no difference in UACR between subjects in different tertiles. The proportion of CKD showed a significant difference among the three group(p<0.05).

**Table 1:** Clinical features of the whole population stratified across tertiles of renal FF measured by the MRI.

	All	Tertile 1 (2.498-3.778)	Tertile 2 (3.779 - 4.690)	Tertile 3 (4.691-7.434)	*p*-value
	n = 189	n = 63	n = 63	n = 63	
Male,n (%)	125 (66.1)	39 (61.9)	46 (73.0)	40 (63.5)	0.362
Age (years)	57 ± 11	54 ± 12	58 ± 10	59 ± 10	0.041
Duration of diabetes (years)	10 ± 7	9 ± 7	11 ± 8	11 ± 7	0.412
Smoking history,n (%)	85 (45.0)	33 (52.4)	28 (44.4)	24 (38.1)	0.271
Drinking history,n (%)	55 (29.1)	16 (25.4)	19 (30.2)	20 (31.7)	0.716
History of hypertension, n (%)	92 (48.7)	30 (47.6)	29 (46.0)	33 (52.4)	0.759
SBP (mmHg)	132 ± 17	136 ± 19	131 ± 17	129 ± 14	0.061
DBP (mmHg)	81 ± 10	83 ± 11	79 ± 9	81 ± 9	0.092
BMI (kg/m2)	25.3 ± 3.0	24.9 ± 3.1	25.2 ± 2.7	25.9 ± 3.3	0.157
WC (cm)	93.2 ± 8.1	91.0 ± 8.6	92.3 ± 6.6	96.2 ± 8.1	0.001
HIP (cm)	96.5 ± 6.2	96.3 ± 6.0	95.6 ± 5.8	97.7 ± 6.7	0.171
FPG (mmol/L)	9.2 ± 3.9	9.9 ± 4.7	8.8 ± 3.3	9 ± 3.4	0.271
HbA1c (mmol/mol)	8.5 ± 2.2	8.9 ± 2.3	8.3 ± 2.0	8.4 ± 2.3	0.362
TC (mmol/L)	4.49 ± 1.35	4.59 ± 1.38	4.44 ± 1.29	4.43 ± 1.38	0.750
TG (mmol/L)	2.53 ± 2.82	2.40 ± 2.55	2.39 ± 2.46	2.81 ± 3.37	0.635
HDL-C (mmol/L)	1.10 ± 0.31	1.10 ± 0.30	1.09 ± 0.33	1.09 ± 0.31	0.959
LDL-C (mmol/L)	2.57 ± 1.01	2.72 ± 1.09	2.72 ± 1.09	2.48 ± 1.01	0.367
UACR (mg/g)	204.1 ± 669.5	158.9 ± 630.1	209.3 ± 621.7	244.2 ± 756.3	0.774
eGFR (ml/min/1.73 m^2^)	87.7 ± 23.0	97.2 ± 19.8	85.8 ± 23.5	80.1 ± 22.5	0.000
CKD,n (%)	32 (16.9)	5 (7.9)	11 (17.5)	16 (25.4)	0.033
Insulin, n (%)	73 (39.7)	19 (31.7)	31 (50.0)	23 (37.1)	0.103
RX with ACE-I/ARBs, n (%)	60 (31.7)	18 (28.6)	22 (34.9)	20 (31.7)	0.746
Hypolipidemic therapy, n (%)	73 (38.6)	20 (31.7)	28 (44.4)	25 (39.7)	0.335
Renal FF (%)	4.19 (3.59,5.03)	3.44 (3.12,3.59)	4.20 (4.04,4.52)	5.30 (5.01,5.9)	0.000

SBP, systolic blood pressure; DBP, diastolic blood pressure; BMI, body mass index; WC, waist circumference; HIP, hip circumference; FPG, Fasting plasma glucose; HbA1c, glycated hemoglobin; TG, total triglyceride; TC, total cholesterol; HDL-C, high densitylipoprotein cholesterol; LDL-C, low density lipoprotein cholesterol; UACR, urinary microalbumin creatinine ratio; eGFR, estimated Glomerular Filtration Rate; CKD, chronic kidney disease; ACEI, angiotensin converting enzyme inhibitors; ARB, angiotensin receptor antagonists; FF, fat fraction.

### Comparison of fat parameters between CKD and non-CKD

Of the 189 participants, 32 (16.9%) had CKD. Participants with CKD had a higher renal FF than patients without [ 4.69%(4.19%, 5.39%) vs. 4.08% (3.55%, 4.97%), p = 0.005]. No significant intergroup differences were detected with respect to BMI and WC. The results are shown in **Table 2**.

**Table 2:** The differences of BMI, WC and renal FF parameters between non-CKD group (eGFR ≥ 60ml/min/1.73m^2^) and CKD group (eGFR < 60ml/min/1.73m^2^).

	non-CKD	CKD	*p*-value
	n = 156	n = 33	
BMI (kg/m^2^)	25.2 ± 3.0	25.8 ± 3.2	0.335
WC (cm)	92.8 ± 8.0	95.0 ± 8.2	0.142
Renal FF	4.3 ± 1.03	4.78 ± 0.89	0.014
L-Renal FF	2.36 ± 0.64	2.52 ± 0.63	0.215
R-Renal FF	1.94 ± 0.54	2.27 ± 0.45	0.001

BMI, body mass index; WC, waist circumference; FF, fat fraction; L-Renal FF, renal fat fraction on the left; R-Renal FF, renal fat fraction on the right; CKD, chronic kidney disease; eGFR, estimated Glomerular Filtration Rate.

### Relationship between renal FF and CKD

**Figure 3:**
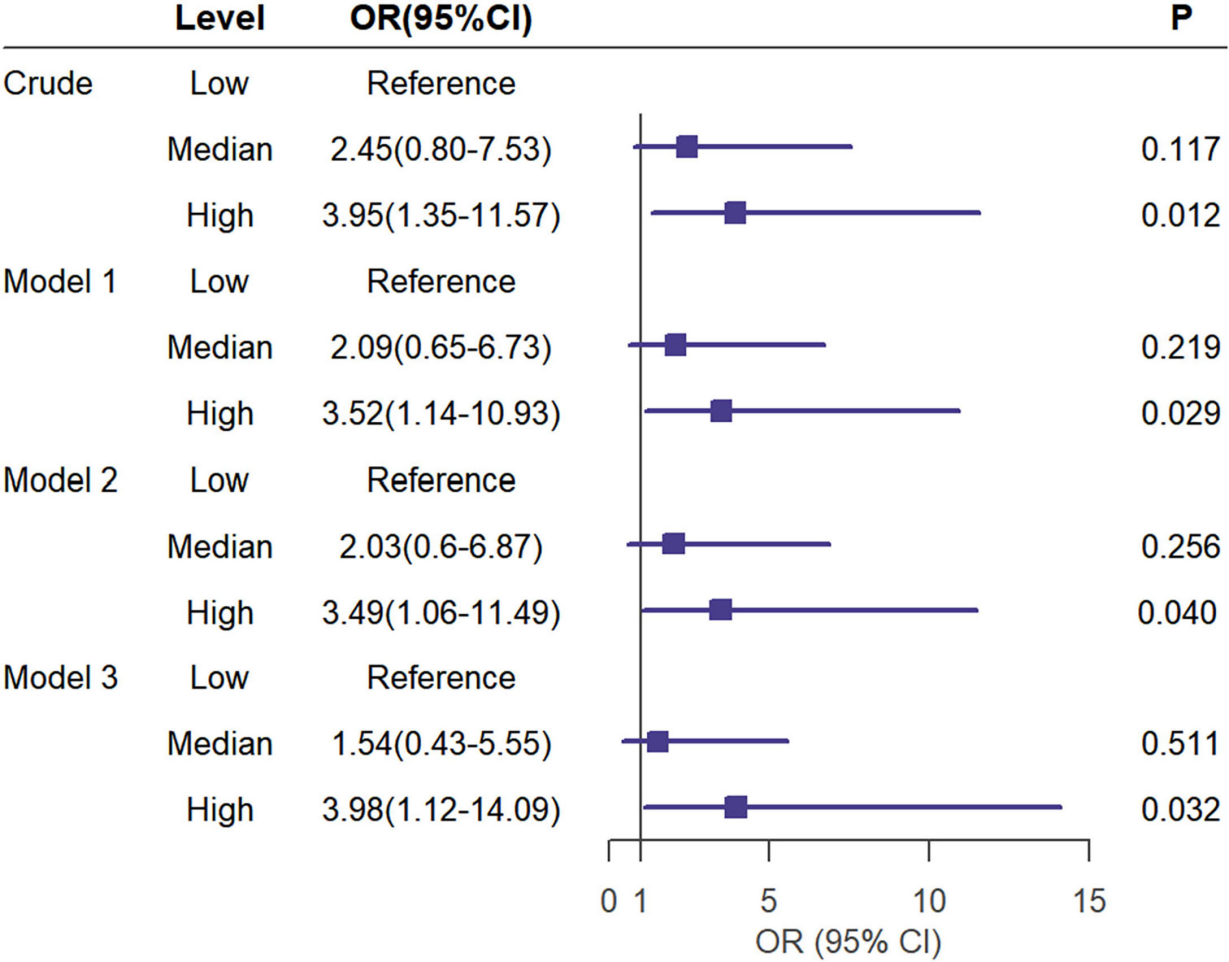
Univariate (crude model) and multivariate analyses (Model 1 to 3) for logistic regression of CKD(eGFR < 60ml/min/1.73m2) risk according to the tertiles of the renal FF. Model 1 was adjusted for age and sex. Model 2 was adjusted for the BMI,WC,HbA1C,TG in addition to the variables in model 1. Model 3 was adjusted for duration of diabetes mellitus, history of hypertension,smoking history,drinking history,insulin therapy, RX with ACE-I/ARBs, hypolipidemic therapy in addition to the variables in model 2.

**Figure 4:**
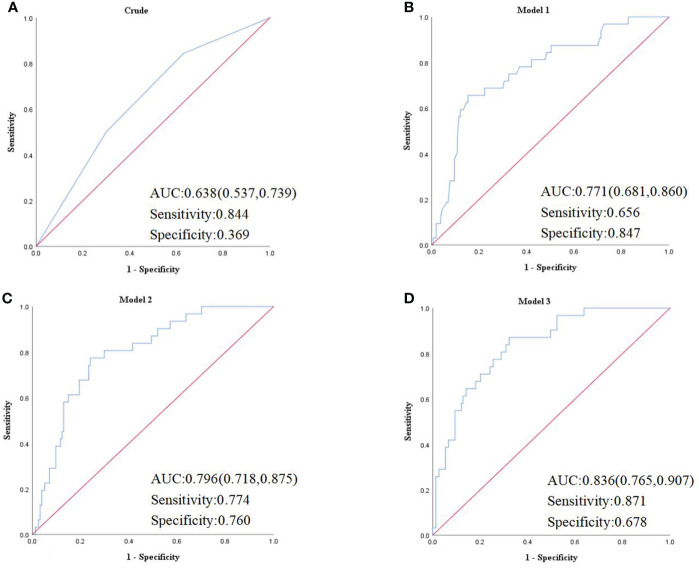
**(A)** Crude model was unadjusted. **(B) **Model 1 was adjusted for age and sex. **(C)** Model 2 was adjusted for the BMI, WC, HbA1C, TG, UACR in addition to the variables in model 1. **(D)** Model 3 was adjusted for duration of diabetes mellitus, history of hypertension, smoking history, drinking history, insulin therapy, RX with ACE-I/ARBs, hypolipidemic therapy in addition to the variables in model 2. AUC, area under the curve.

The binary logistic regression analysis was used to assess the correlation of the renal FF with CKD. In the crude model, with the low tertile of the renal FF set as the reference, the renal FF in the top tertile was associated with a higher OR for CKD (OR = 3.95, 95% CI =1.35 - 11.57, p = 0.012). After adjustment for sex and age, renal FF located in the high-quartet still had a significantly increased risk of CKD relative to the low-quartet (OR = 3.52, 95% CI = 1.14 - 10.93, p = 0.029). After additional adjustment for BMI, WC, HbA1C, and TG (model 2), plus DM duration, history of hypertension, smoking and drinking history, insulin therapy, anti-hypertension drugs, and hypoglycemic therapy (model 3), this trend was retained (**Figure 3**). The area under the ROC curve was 0.836 (range, 0.765–0.907), with sensitivity of 0.871 and specificity of 0.678 (**Figure 4**).

## Discussion

Our research studied the relationship between renal FF measured by MRI and CKD in patients with T2DM, and demonstrated that increased renal FF was an independent risk factor for CKD after controlling for other possible confounding variables.

Previous animal studies (28, 29) have shown that renal lipid deposition is closely related to the development of kidney damage. Owing to the lack of effective measurement methods, few population studies on the relationship between renal parenchymal fat deposits and diabetes. A cross-sectional study (30) enrolled 29 patients with T2DM and 40 non-diabetic patients in USA, using MRI to access renal FF of patients, and found that renal FF in patients with T2DM was significantly increased compared with the control group, and T2DM was significantly associated with renal FF after adjusting for age, sex, BMI, and hepatic FF. Another cross-sectional study (23) included 95 subjects and divided them into three groups, non-diabetic volunteers(n=34), patients with T2DM with normoalbuminuria (n=21) and patients with T2DM with microalbuminuria (n=40). After comparing the renal FF of patients, they found renal FF of patients with T2DM with microalbuminuria is significantly increased. However, this study failed to further analyze its relationship with kidney function. Our cross-sectional study enrolled 189 subjects with averaged eGFR 87.7 mL/min/1.73 m^2^, found that high FF increase the risk of CKD, the area under the ROC curve was 0.836. Two experienced radiology doctors measured renal parenchymal fat by selecting 5 sections of each kidney, and renal FF was obtained by adding up the average of bilateral renal FF in each individual. The consistency within the group was good (ICC = 0.822). It is suggested that renal parenchymal fat is an independent risk factor for CKD.

Some studies (21, 31) have shown that there are abnormalities in kidney fat metabolism in diabetic patients, such as decreased β-oxidation of fatty acids, and increased intake of cholesterols and fatty acids, which is one of the main mechanisms of kidney fat deposition in patients with T2DM. The possible mechanism for kidney dysfunction caused by renal FF is lipotoxicity. Excessive lipids accumulate in the renal parenchyma, causing damage to various cells, including podocytes (16), mesangial cells (17), and proximal tubular epithelial cells (18, 19),being substantially detrimental to renal function in the long term. Besides, lipids accumulation within the renal parenchyma may cause oxidative stress, inflammation, and fibrosis (32, 33) and lead to renal damage.

Our study had some limitations. First. this was a cross-sectional study so that we could not establish a causal effect relationship between renal FF and CKD. Indeed, our study is the first to explore the relationship between renal fat and CKD in patients with T2DM, which is innovative. Second, the sample size of our study was relatively small because of the high expense of MRI. Nevertheless, our method of measuring renal parenchymal fat deposition is noninvasive and accurate compared to other methods, and our findings are credible. Further longitudinal and larger sample sizes studies are needed to ensure the association between the renal FF and CKD in diabetic patients.

## Conclusions

The present study demonstrates that increased renal FF is significantly associated with CKD in patients with T2DM, independent of anthropometric, metabolic factors, and other fat indicators. Renal FF may be a reliable and valuable anthropometric measurement for the early identification of risk of CKD in patients with T2DM.

## Data availability statement

The original contributions presented in the study are included in the article/Supplementary Material. Further inquiries can be directed to the corresponding authors.

## Ethics statement

The studies involving human participants were reviewed and approved by the Ethics Committee of The First Affiliated Hospital of Chongqing Medical University. The patients/participants provided their written informed consent to participate in this study. Written informed consent was obtained from the individual(s) for the publication of any potentially identifiable images or data included in this article.

## Author contributions

All authors made a significant contribution to the work reported by participating in some or all of the following processes: conception, design and execution of study; acquisition, analysis and interpretation of data; and drafting, revising or critically reviewing the article. All authors gave final approval of the version to be published, agreed on the journal to which the article has been submitted and agreed to be accountable for all aspects of the work.

## Funding

This work was supported by the Technological Innovation and Application Development Project of Chongqing (cstc2019jscx-msxmX0207), the Chongqing Science and Health Joint Medical Research Project (2020FYYX141; 2018GDRC004), the Innovative Funded Project of Chongqing Innovation and Retention Program (cx2019032) and the Chongqing Yong and Middle-aged Senior Medical Talents Studio (ZQNYXGDRCGZS2021001) and the National Natural Science Foundation of China (81870567).

## Conflict of interest

The authors declare that the research was conducted in the absence of any commercial or financial relationships that could be construed as a potential conflict of interest.

## Publisher’s note

All claims expressed in this article are solely those of the authors and do not necessarily represent those of their affiliated organizations, or those of the publisher, the editors and the reviewers. Any product that may be evaluated in this article, or claim that may be made by its manufacturer, is not guaranteed or endorsed by the publisher.

## References

[1] Bramlage P Lanzinger S van Mark G Hess E Fahrner S Heyer CHJ. Patient and disease characteristics of type-2 diabetes patients with or without chronic kidney disease: an analysis of the German DPV and DIVE databases. Cardiovasc Diabetol (2019) 18(1):33. doi: 10.1186/s12933-019-0837-x 30878037 PMC6420726

[2] Low SK Sum CF Yeoh LY Tavintharan S Ng XW Lee SB. Prevalence of chronic kidney disease in adults with type 2 diabetes mellitus. Ann Acad Med Singap (2015) 44(5):164–71. doi: 10.47102/annals-acadmedsg.V44N5p164 26198322

[3] Guo K Zhang L Zhao F Lu J Pan P Yu H. Prevalence of chronic kidney disease and associated factors in Chinese individuals with type 2 diabetes: Cross-sectional study. J Diabetes Complications (2016) 30(5):803–10. doi: 10.1016/j.jdiacomp.2016.03.020 27068269

[4] MacIsaac RJ Jerums G Ekinci EI. Effects of glycaemic management on diabetic kidney disease. World J Diabetes (2017) 8(5):172–86. doi: 10.4239/wjd.v8.i5.172 PMC5437616 28572879

[5] Vanholder R Massy Z Argiles A Spasovski G Verbeke F Lameire N. Chronic kidney disease as cause of cardiovascular morbidity and mortality. Nephrol Dial Transplant (2005) 20(6):1048–56. doi: 10.1093/ndt/gfh813 15814534

[6] Fu H Liu S Bastacky SI Wang X Tian XJ Zhou D. Diabetic kidney diseases revisited: A new perspective for a new era. Mol Metab (2019) 30:250–63. doi: 10.1016/j.molmet.2019.10.005 PMC6838932 31767176

[7] Cheng HT Xu X Lim PS Hung KY. Worldwide epidemiology of diabetes-related end-stage renal disease, 2000-2015. Diabetes Care (2021) 44(1):89–97. doi: 10.2337/dc20-1913 33203706

[8] Afkarian M Sachs MC Kestenbaum B Hirsch IB Tuttle KR Himmelfarb J. Kidney disease and increased mortality risk in type 2 diabetes. J Am Soc Nephrol (2013) 24(2):302–8. doi: 10.1681/ASN.2012070718 PMC3559486 23362314

[9] Hager MR Narla AD Tannock LR. Dyslipidemia in patients with chronic kidney disease. Rev Endocr Metab Disord (2017) 18(1):29–40. doi: 10.1007/s11154-016-9402-z 28000009

[10] Choi SR Lim JH Kim MY Kim EN Kim Y Choi BS. Adiponectin receptor agonist AdipoRon decreased ceramide, and lipotoxicity, and ameliorated diabetic nephropathy. Metabolism (2018) 85:348–60. doi: 10.1016/j.metabol.2018.02.004 29462574

[11] Ertunc ME Hotamisligil GS. Lipid signaling and lipotoxicity in metaflammation: indications for metabolic disease pathogenesis and treatment. J Lipid Res (2016) 57(12):2099–114. doi: 10.1194/jlr.R066514 PMC5321214 27330055

[12] Mende CW Einhorn D. Fatty kidney disease: A new renal and endocrine clinical entity? describing the role of the kidney in obesity, metabolic syndrome, and type 2 diabetes. Endocr Pract (2019) 25(8):854–8. doi: 10.4158/EP-2018-0568 31013163

[13] Chen X Mao Y Hu J Han S Gong L Luo T. Perirenal fat thickness is significantly associated with the risk for development of chronic kidney disease in patients with diabetes. Diabetes (2021) 70(10):2322–32. doi: 10.2337/db20-1031 34593536

[14] Spit KA Muskiet MHA Tonneijck L Smits MM Kramer MHH Joles JA. Renal sinus fat and renal hemodynamics: a cross-sectional analysis. MAGMA (2020) 33(1):73–80. doi: 10.1007/s10334-019-00773-z 31471702 PMC7021744

[15] Foster MC Hwang SJ Porter SA Massaro JM Hoffmann U Fox CS. Fatty kidney, hypertension, and chronic kidney disease: the framingham heart study. Hypertension (2011) 58(5):784–90. doi: 10.1161/HYPERTENSIONAHA.111.175315 PMC3204377 21931075

[16] D'Agati VD Chagnac A de Vries AP Levi M Porrini E Herman-Edelstein M. Obesity-related glomerulopathy: clinical and pathologic characteristics and pathogenesis. Nat Rev Nephrol (2016) 12(8):453–71. doi: 10.1038/nrneph.2016.75 27263398

[17] Li J Li H Wen YB Li XW. Very-low-density lipoprotein-induced triglyceride accumulation in human mesangial cells is mainly mediated by lipoprotein lipase. Nephron Physiol (2008) 110(1):p1–10. doi: 10.1159/000151272 18698144

[18] Kang HM Ahn SH Choi P Ko YA Han SH Chinga F. Defective fatty acid oxidation in renal tubular epithelial cells has a key role in kidney fibrosis development. Nat Med (2015) 21(1):37–46. doi: 10.1038/nm.3762 25419705 PMC4444078

[19] Vaziri ND. Disorders of lipid metabolism in nephrotic syndrome: mechanisms and consequences. Kidney Int (2016) 90(1):41–52. doi: 10.1016/j.kint.2016.02.026 27165836 PMC5812444

[20] Sun L Halaihel N Zhang W Rogers T Levi M. Role of sterol regulatory element-binding protein 1 in regulation of renal lipid metabolism and glomerulosclerosis in diabetes mellitus. J Biol Chem (2002) 277(21):18919–27. doi: 10.1074/jbc.M110650200 11875060

[21] Wang Z Jiang T Li J Proctor G McManaman JL Lucia S. Regulation of renal lipid metabolism, lipid accumulation, and glomerulosclerosis in FVBdb/db mice with type 2 diabetes. Diabetes (2005) 54(8):2328–35. doi: 10.2337/diabetes.54.8.2328 16046298

[22] Reeder SB Hu HH Sirlin CB. Proton density fat-fraction: a standardized MR-based biomarker of tissue fat concentration. J Magn Reson Imaging (2012) 36(5):1011–4. doi: 10.1002/jmri.23741 PMC4779595 22777847

[23] Wang YC Feng Y Lu CQ Ju S. Renal fat fraction and diffusion tensor imaging in patients with early-stage diabetic nephropathy. Eur Radiol (2018) 28(8):3326–34. doi: 10.1007/s00330-017-5298-6 29450711

[24] Alberti KG Zimmet PZ. Definition, diagnosis and classification of diabetes mellitus and its complications. part 1: diagnosis and classification of diabetes mellitus provisional report of a WHO consultation. Diabetes Med (1998) 15(7):539–53. doi: 10.1002/(SICI)1096-9136(199807)15:7<539::AID-DIA668>3.0.CO;2-S 9686693

[25] Ma YC Zuo L Chen JH Luo Q Yu XQ Li Y. Modified glomerular filtration rate estimating equation for Chinese patients with chronic kidney disease. J Am Soc Nephrol (2006) 17(10):2937–44. doi: 10.1681/ASN.2006040368 16988059

[26] American Diabetes Association Professional Practice, Committee Draznin B Aroda VR Bakris G Benson G Brown FM. Chronic kidney disease and risk management: Standards of medical care in diabetes-2022. Diabetes Care (2022) 45(Suppl 1):S175–84. doi: 10.2337/dc22-S011 34964873

[27] Koo TK Li MY. A guideline of selecting and reporting intraclass correlation coefficients for reliability research. J Chiropr Med (2016) 15(2):155–63. doi: 10.1016/j.jcm.2016.02.012 PMC4913118 27330520

[28] de Vries AP Ruggenenti P Ruan XZ Praga M Cruzado JM Bajema IM. Fatty kidney: emerging role of ectopic lipid in obesity-related renal disease. Lancet Diabetes Endocrinol (2014) 2(5):417–26. doi: 10.1016/S2213-8587(14)70065-8 24795255

[29] DeZwaan-McCabe D Sheldon RD Gorecki MC Guo DF Gansemer ER Kaufman RJ. ER stress inhibits liver fatty acid oxidation while unmitigated stress leads to anorexia-induced lipolysis and both liver and kidney steatosis. Cell Rep (2017) 19(9):1794–806. doi: 10.1016/j.celrep.2017.05.020 PMC5520660 28564599

[30] Yokoo T Clark HR Pedrosa I Yuan Q Dimitrov I Zhang Y. Quantification of renal steatosis in type II diabetes mellitus using dixon-based MRI. J Magn Reson Imaging (2016) 44(5):1312–9. doi: 10.1002/jmri.25252 PMC5035175 27007212

[31] Herman-Edelstein M Scherzer P Tobar A Levi M Gafter U. Altered renal lipid metabolism and renal lipid accumulation in human diabetic nephropathy. J Lipid Res (2014) 55(3):561–72. 10.1194/jlr.P040501 PMC3934740 24371263

[32] Ashcroft FM Rohm M Clark A Brereton MF. Is type 2 diabetes a glycogen storage disease of pancreatic beta cells? Cell Metab (2017) 26(1):17–23. doi: 10.1016/j.cmet.2017.05.014 28683284 PMC5890904

[33] Thongnak L Pongchaidecha A Lungkaphin A. Renal lipid metabolism and lipotoxicity in diabetes. Am J Med Sci (2020) 359(2):84–99. doi: 10.1016/j.amjms.2019.11.004 32039770

